# FPIRST: Fatigue Driving Recognition Method Based on Feature Parameter Images and a Residual Swin Transformer

**DOI:** 10.3390/s24020636

**Published:** 2024-01-19

**Authors:** Weichu Xiao, Hongli Liu, Ziji Ma, Weihong Chen, Jie Hou

**Affiliations:** 1 College of Electrical and Information Engineering, Hunan University, Changsha 410082, China; Weichuxiao@hnu.edu.cn (W.X.); zijima@hnu.edu.cn (Z.M.); jiehou313@hnu.edu.cn (J.H.); 2 College of Information and Electronic Engineering, Hunan City University, Yiyang 413046, China; 3 College of Information Technology and Management, Hunan University of Finance and Economics, Changsha 410205, China; whchen@hnu.edu.cn

**Keywords:** fatigue driving recognition, facial key points, swin transformer, feature parameter image

## Abstract

Fatigue driving is a serious threat to road safety, which is why accurately identifying fatigue driving behavior and warning drivers in time are of great significance in improving traffic safety. However, accurately recognizing fatigue driving is still challenging due to large intra-class variations in facial expression, continuity of behaviors, and illumination conditions. A fatigue driving recognition method based on feature parameter images and a residual Swin Transformer is proposed in this paper. First, the face region is detected through spatial pyramid pooling and a multi-scale feature output module. Then, a multi-scale facial landmark detector is used to locate 23 key points on the face. The aspect ratios of the eyes and mouth are calculated based on the coordinates of these key points, and a feature parameter matrix for fatigue driving recognition is obtained. Finally, the feature parameter matrix is converted into an image, and the residual Swin Transformer network is presented to recognize fatigue driving. Experimental results on the HNUFD dataset show that the proposed method achieves an accuracy of 96.512%, thus outperforming state-of-the-art methods.

## 1. Introduction

Fatigue driving leads to a decline in driving skills due to the imbalance between the physical and psychological functions of drivers during long-term driving, and is the main cause of traffic accidents. In the United States, more than 100,000 traffic accidents are caused by fatigue driving every year, of which more than 7 million people are injured or killed [1]. French traffic accident statistics show that fatigue driving accounts for about 15% of all injuries and 21% of deaths [2]. According to the Ministry of Communications of China, traffic accidents caused by fatigue driving accounted for about 20% of total accidents, 40% of major traffic accidents, and 83% of traffic-related deaths [3]. However, if drivers are warned half a second in advance, about 60% of traffic accidents can be effectively avoided. Therefore, accurately recognizing the driver’s fatigue driving status and warning them promptly are urgent matters, making them research hotspots in the field of traffic safety.

Existing vision-based fatigue driving recognition methods can usually be summarized in three steps: face detection, facial feature extraction, and fatigue state decision [4]. Drivers’ face detection methods include multi-task convolutional neural networks (MTCNNs) [5], multi-scale feature output, and spatial pyramid pooling [6]. Extracting facial fatigue features includes blinking and yawning, which are usually described by extracting facial key points. The goal of facial key points is to obtain facial key point coordinates, and methods include the DLIB machine learning toolbox, practical facial landmark detectors (PFLDs) [7], and multi-scale facial landmark detectors [6]. Fatigue status decision methods consist of a statistical threshold, adaptive threshold [6], support vector machine (SVM) [8], long short-term memory (LSTM) network [9], and bidirectional LSTM (Bi-LSTM) [10]. These methods aim to determine whether the tested video indicates fatigue driving. However, these methods are not enough to improve the accuracy of fatigue driving recognition because of the following reasons:

(1) Different drivers show different facial features. Judging whether the driver is fatigued by using a fixed statistical threshold is not a universal approach. The threshold method for fatigue driving recognition necessitates calculating an adaptive threshold for each driver in their normal driving state in advance. However, determining whether their current driving state is their normal driving state is difficult in practical applications.
(2) The SVM method judges fatigue driving through data classification. The computational complexity of this method depends on the number of support vectors, and predictive time is proportional to the number of support vectors. Thus, it is more sensitive to missing data. Finding a suitable nuclear function to transform data dimensions is difficult, thus affecting the accuracy of classification.
(3) The LSTM network selectively remembers or forgets information through gating units. It learns to enter long-term dependencies in the sequence, effectively controlling the flow and outflow of information, and passes this information to the next time step. However, it needs improvement in accurately capturing important information in the input sequence.

This paper proposes a novel fatigue driving recognition (FPIRST) method for complex driving scenarios to improve recognition accuracy. The proposed method takes advantage of facial key points to compute feature parameter values and formulate feature parameter matrices and images. Furthermore, feature parameter images are input into a residual Swin Transformer network for fatigue driving recognition. The main contributions of this study are summarized as follows:

(1) A fatigue driving recognition framework based on feature parameter images and a residual Swin Transformer is designed. In the FPIRST, SPP-MSFO is used to detect the face region, and MSFLD is adopted to locate facial key points. On the basis of the key points, the feature parameter images are formulated, and the residual Swin Transformer network is used to recognize fatigue driving.
(2) The aspect ratios of the mouth, left eye, and right eye are computed based on facial 23 key point coordinates to formulate feature parameter images. The feature parameter matrix of *n* × 3 can be obtained from *n*-frame images. Subsequently, the technique of sliding *k* frames is used to expand the *n* × 3 feature parameter matrix into an *m* 224 × 224 matrix. Each feature parameter matrix of 224 × 224 is converted into a feature parameter image. Such images contain not only the characteristics of feature parameters but also the duration information of fatigue driving behavior.
(3) A residual Swin Transformer module is used to recognize fatigue driving behavior. The residual Swin Transformer can represent features more compactly and obtain richer semantic information, therefore better locating targets. The skip connection in the residual Swim Transformer realizes selective multi-scale learning of local discriminative features in diving video sequences. The experimental results on the HNUFD dataset verify the proposed method.

## 2. Related Work

### 2.1. Fatigue Driving Recognition Methods

Fatigue driving recognition includes physiological feature-based, vision-based, and hybrid methods [11]. With the development of computer vision technology, the vision-based fatigue driving recognition method has become the mainstream method. In a vision-based fatigue driving recognition system, a vehicle-mounted camera is placed in the right front of the cab to capture the driver’s state in real time. Puspasari et al. proposed a fatigue driving recognition method based on SVM, which uses radial basis function as the kernel function of SVM to identify fatigue driving status [8]. Zhang et al. proposed a fatigue driving recognition method based on facial key points [12]. This method calculates the aspect ratio of the eyes, the aspect ratio of the mouth, and the rotation angle of the head according to the facial key points in the Dlib library. The states of eye closure, yawning, and lowered head are detected by setting a fixed threshold. Chen et al. proposed a fatigue driving detection method based on facial key points and an LSTM network [9]. This method first uses the MTCNN for face detection. Then, the Dlib library is used to locate the facial key points of each frame image and extract the fatigue feature vector. Finally, the information group of multiple continuous frames is synthesized into a time feature sequence, which is sent to the LSTM network to identify the fatigue driving state. Hu et al. proposed a hybrid method of fatigue driving recognition based on a 3D conditional generative adversarial network and a two-level attention Bi-LSTM network [10]. First, MTCNN is used to capture facial regions from original videos, and then short-term fatigue-related information is learned through a 3D conditional generative adversarial network. Then, the long-term spatial-temporal representation is learned through the two-level attention Bi-LSTM network. Finally, the results of fatigue driving are predicted by temporal smoothing. Xiao et al. proposed a fatigue driving recognition method based on MSFLD [6]. The method first adopts the SPP-MSFO model to detect the face region and then locates 23 key points on the face through MSFLD. Then, the aspect ratios of the mouth, left eye, and right eye are calculated according to the key points, and a fatigue parameter matrix is formed. Finally, the method combining adaptive threshold and statistical threshold is adopted to identify the fatigue driving state.

### 2.2. Image Classification Methods

Image classification has always been a hot research direction, and the emergence of deep learning has promoted the development of this field. At present, image classification methods include the feature extraction-based method [13,14,15] and the deep learning (DL) method [16,17], in which the DL method mainly include convolution neural networks (CNNs) and Transformer.

The classification of fatigue EEG signals uses SVM, and the result is taken as the initial fatigue value [13]. The multi-view learning method adopts double-sided twin SVM to extract features for binary classification [14]. The discriminant subspace (RDS) learning method is used for feature extraction to promote the robustness of the models [15]. The within-class distances are measured based on L2,s-norm, and the between-class distances are measured based on L2,p-norm.

The image classification methods based on CNN mainly include LeNet, AlexNet, VGGNet, GoogLeNet, and residual learning networks (ResNets). Lecun et al. first applied LeNet CNNs for image classification, achieving great success in handwritten digit recognition tasks [18]. LeNet extracts image features by continuously using a combination structure of convolution, pooling, and nonlinear mapping, and then calculates the prediction probability for each category through the activation function Softmax. Krizhevsky et al. proposed an AlexNet network for image classification [19]. AlexNet has a deeper network structure than LeNet, consisting of five convolutional layers and three full connection layers. Simonyan et al. proposed a VGG network for large-scale image classification [20]. VGG uses a series of convolution kernels with a size of 3 × 3 and the pooling layer to construct the depth of CNNs. It explores the relationship between network depth and performance and achieves good results. Szegedy et al. proposed a deep network called GoogLeNet based on an Inception structure for image classification [21]. The inception module operates on the input image through three convolution kernels of different sizes and maximum pooling. Then, the outputs of these four operations are spliced along the channel to form an output feature map. It contains features extracted from convolution kernels of different sizes, which capture multi-scale feature information. With the deepening of the number of network layers, the performance of deep learning networks can be improved. However, the existence of nonlinear activation functions means that when the network deepens to a certain extent, it will cause considerable irreversible information loss, which is called a network degradation problem. To address this problem, He et al. proposed ResNet [22]. ResNet aims to solve the problem of network degradation by introducing a deep residual learning framework so that the network can perform identity mapping.

The image classification methods based on Transformer mainly include Vision Transformer (ViT) and Swin Transformer. Dosovitskiy et al. proposed a ViT method for image classification [23]. In ViT, an image is divided into fixed-size patches, and the linear embedding sequences of these patches are input into the Transformer Encoder. Meanwhile, the multilayer perceptron head is used for image classification. Liu et al. proposed a Swin Transformer method for image classification [24]. The Swin Transformer method has two improvements over ViT: (1) A hierarchical Transformer is established, which enables the features of different layers to have different meanings. The shallow layer features have large-scale and detailed information, and the deep layer features have small-scale and overall outline information. (2) The idea of locality is introduced to conduct self-attention calculation in the region of the non-coincidence window. It not only reduces the computation amount but also increases the interaction between different windows.

## 3. Proposed Method

In this section, the overall structure of the proposed method is presented, the feature parameter matrix and images are built, and the residual Swim Transformer network is proposed for fatigue driving recognition.

### 3.1. Overview of the Architecture

**Figure 1:**
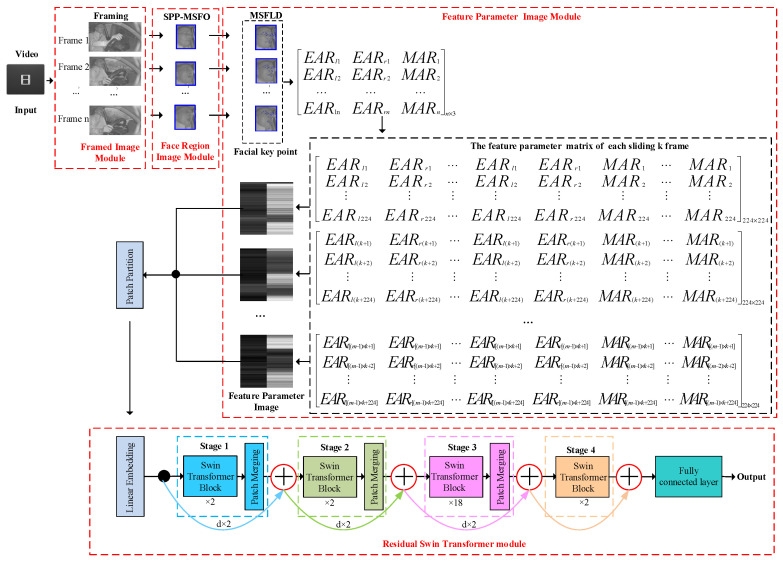
Architecture overview diagram of the proposed method.

The overview of the proposed method is shown in Figure 1. The FPIRST method is based on a feature parameter image and a residual Swin Transformer, which consists of a framed image module, face region image module, feature parameter image module, and residual Swin Transformer module. First, the frame image module divides the video into images. Second, the face region image is detected using the SPP-MSFO module. Third, MSFLD is used to locate 23 key points, and the coordinates of these points are obtained. Fourth, the aspect ratios of the eyes and mouth are calculated based on the coordinates of these key points, and a feature parameter matrix with a size of *n* × 3 is formed. In addition, the matrix is expanded from *n* × 3 to 224 × 224 by using the technique of sliding k frames. Such matrices are converted into images, and the feature parameter images are obtained. Finally, the feature parameter images are passed to the residual Swin Transformer classifier and the fatigue driving behavior of the input video is identified.

### 3.2. Feature Parameter Image Module

Based on the detection results of facial key points, the proposed method extracts the features of the eyes and mouth to obtain the feature parameter matrix. Then, the matrix is converted into feature parameter images by using sliding *k* and filling techniques.

#### 3.2.1. Feature Extraction of Eye Fatigue

**Figure 2:**
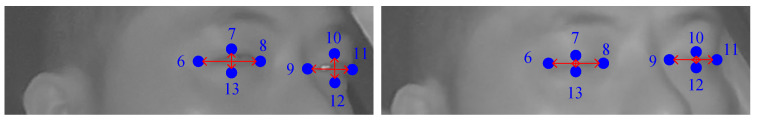
Diagram of eye-opening and closing states.

The degree of eye closure is an important feature of fatigue driving, which can be used to judge whether the driver is dozing off or not. When people open their eyes, the distance between the upper and lower feature points of the eyes will become larger, and it becomes smaller when the eyes are closed. Figure 2 shows the states of opening and closing the eyes, where the number is the index of key points. In this paper, the left-eye aspect ratio *EAR_l_* and the right-eye aspect ratio *EAR_r_* are used to judge the driver’s eye-opening and closing state. *EAR_l_* and *EAR_r_* are calculated by Equations (1) and (2), respectively.

(1) $$EAR_{l}=\frac{y_{13}-y_{7}}{x_{8}-x_{6}},$$

(2) $$EAR_{r}=\frac{y_{12}-y_{10}}{x_{11}-x_{9}},$$

where $x_{6}$ , $x_{8}$ , $x_{9}$ , and $x_{11}$ are the abscissas of the key points of the left eye and the right eye, respectively. $y_{7}$ , $y_{13}$ , $y_{10}$ , and $y_{12}$ are the vertical coordinates of the key points of the left eye and the right eye, respectively.

#### 3.2.2. Feature Extraction of Mouth Fatigue

**Figure 3:**
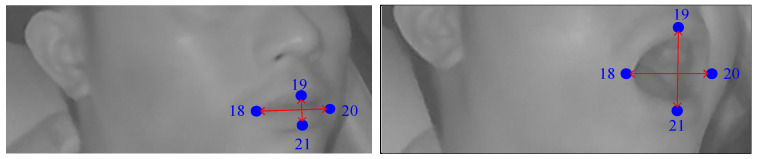
Diagram of mouth-closing and yawning states.

The degree of mouth opening is also an important feature of fatigue driving and can determine whether the driver is yawning. When the driver is yawning, the opening of the mouth widens. At this time, the height between the upper and lower feature points of the mouth increases, which is higher than that of normal driving. Meanwhile, the width between the left and right feature points of the mouth decreases, which is lower than that of normal driving. In contrast, when the mouth opens during speaking or closes, the height between the upper and lower feature points of the mouth is small. Figure 3 shows the states of closed mouth and yawning, where the number is the index of key points. In this paper, the mouth aspect ratio *MAR* is used to judge the degree of mouth opening, which is calculated in Equation (3):

(3) $$MAR=\frac{y_{21}-y_{19}}{x_{20}-x_{18}},$$

where $x_{18}$ and $x_{20}$ are the abscissas of the two key points on the left and right of the mouth, and $y_{19}$ and $y_{21}$ are the ordinates of the two key points above and below the mouth.

#### 3.2.3. Generating the Feature Parameter Matrix of n×3

The feature parameter vector $F_{i}$ of a single frame image is expressed as:

(4) $$F_{i}=(EAR_{li},\;EAR_{ri},\;MAR_{i}\})$$

The feature parameter vector of each frame image has three columns, and the corresponding matrix size is 1 × 3. Fatigue driving is a kind of continuous driving behavior related to time. Thus, for fatigue driving recognition, multiple consecutive frames need to be analyzed rather than a single frame. In this paper, multiple consecutive frames are utilized to generate an n×3 feature parameter matrix *F*, which contains driving behavior time information. The feature parameter matrix is shown in Equation (5), where the first, second, and third columns represent the aspect ratios of the left eye, right eye, and mouth of each frame, respectively.

(5) $$F=\left[\begin{array}{ccc} EAR_{l1} & EAR_{r1} & MAR_{1}\\ EAR_{l2} & EAR_{r2} & MAR_{2}\\ \vdots & \vdots & \vdots \\ EAR_{ln} & EAR_{rn} & MAR_{n} \end{array}\right]$$

#### 3.2.4. Generating Feature Parameter Image of 224×224

Feature parameters are inputted into the residual Swin Transformer classifier by converting the feature parameter matrix into feature parameter images by sliding *k* frames each time and matrix filling techniques. First, for the n×3 feature parameter matrix, $EAR_{l}$ and $EAR_{r}$ in each row are repeated 56 times, and MAR is repeated 112 times, resulting in the feature parameter matrix with a size of n×224 . The feature parameter matrix is described in Equation (6):

(6) $$M1={\left[\begin{array}{cccccccc} EAR_{l1} & EAR_{r1} & \ldots & EAR_{l1} & EAR_{r1} & MAR_{1} & \ldots & MAR_{1}\\ EAR_{l2} & EAR_{r2} & \ldots & EAR_{l2} & EAR_{r2} & MAR_{2} & \ldots & MAR_{2}\\ \vdots & \vdots & \vdots & \vdots & \vdots & \vdots & \vdots & \vdots \\ EAR_{ln} & EAR_{rn} & \ldots & EAR_{ln} & EAR_{rn} & MAR_{n} & \ldots & MAR_{n} \end{array}\right]}_{n\times 224}$$

Then, by sliding k frames each time, the n×224 feature parameter matrix is expanded into m 224 × 224 matrices. *m* is obtained by up-rounding the result of *n* minus 224 divided by *k*, computed as:

(7) $$m=\left\lceil \frac{n-224}{k}\right\rceil$$

The m 224 × 224 matrices are described in Equation (8):

(8) $$T1=\begin{array}{c} {\left[\begin{array}{c} \begin{array}{ccc} {EAR}_{l1} & {EAR}_{r1} & \cdots \\ {EAR}_{l2} & {EAR}_{r2} & \ldots \\ \vdots & \vdots & \vdots \end{array}\;\begin{array}{ccc} {EAR}_{l1} & {EAR}_{r1} & {MAR}_{1}\\ {EAR}_{l2} & {EAR}_{r2} & {MAR}_{2}\\ \vdots & \vdots & \vdots \end{array}\;\begin{array}{cc} \cdots & {MAR}_{1}\\ \ldots & {MAR}_{2}\\ \vdots & \vdots \end{array}\\ \begin{array}{ccc} {EAR}_{l224} & {EAR}_{r224} & \ldots \end{array}\;\begin{array}{ccc} {EAR}_{l224} & {EAR}_{r224} & {MAR}_{224} \end{array}\;\begin{array}{cc} \ldots & {MAR}_{224} \end{array} \end{array}\right]}_{224\times 224}\\ {\left[\begin{array}{c} \begin{array}{ccc} {EAR}_{l(k+1)} & {EAR}_{r(k+1)} & \cdots \\ {EAR}_{l(k+2)} & {EAR}_{r(k+2)} & \ldots \\ \vdots & \vdots & \vdots \end{array}\;\begin{array}{ccc} {EAR}_{l(k+1)} & {EAR}_{r(k+1)} & {MAR}_{\left(k+1\right)}\\ {EAR}_{l(k+2)} & {EAR}_{r(k+2)} & {MAR}_{\left(k+2\right)}\\ \vdots & \vdots & \vdots \end{array}\;\begin{array}{cc} \cdots & {MAR}_{\left(k+1\right)}\\ \ldots & {MAR}_{\left(k+2\right)}\\ \vdots & \vdots \end{array}\\ \begin{array}{ccc} {EAR}_{l(k+224)} & {EAR}_{r(k+224)} & \ldots \end{array}\;\begin{array}{ccc} {EAR}_{l(k+224)} & {EAR}_{r(k+224)} & {MAR}_{\left(k+224\right)} \end{array}\;\begin{array}{cc} \ldots & {MAR}_{\left(k+224\right)} \end{array} \end{array}\right]}_{224\times 224}\\ \begin{array}{c} \vdots \\ {\left[\begin{array}{c} \begin{array}{ccc} {EAR}_{l[(m-1)\times k+1]} & {EAR}_{r[(m-1)\times k+1]} & \cdots \\ {EAR}_{l[(m-1)\times k+2]} & {EAR}_{r[(m-1)\times k+2]} & \ldots \\ \vdots & \vdots & \vdots \end{array}\;\begin{array}{ccc} {EAR}_{l[(m-1)\times k+1]} & {EAR}_{r[(m-1)\times k+1]} & {MAR}_{\left[(m-1)\times k+1\right]}\\ {EAR}_{l[(m-1)\times k+2]} & {EAR}_{r[(m-1)\times k+2]} & {MAR}_{\left[(m-1)\times k+2\right]}\\ \vdots & \vdots & \vdots \end{array}\;\begin{array}{cc} \cdots & {MAR}_{\left[(m-1)\times k+1\right]}\\ \ldots & {MAR}_{\left[(m-1)\times k+2\right]}\\ \vdots & \vdots \end{array}\\ \begin{array}{ccc} {EAR}_{l[(m-1)\times k+224]} & {EAR}_{r[(m-1)\times k+224]} & \ldots \end{array}\;\begin{array}{ccc} {EAR}_{l[(m-1)\times k+224]} & {EAR}_{r[(m-1)\times k+224]} & {MAR}_{\left[(m-1)\times k+224\right]} \end{array}\;\begin{array}{cc} \ldots & {MAR}_{\left[(m-1)\times k+224\right]} \end{array} \end{array}\right]}_{224\times 224} \end{array} \end{array}$$

Finally, the m 224 × 224 matrices are converted into feature parameter images, which are passed into the residual Swin Transformer classifier.

### 3.3. Residual Swin Transformer Module

**Figure 4:**
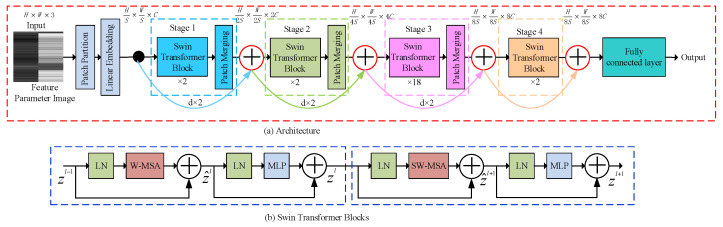
Schematic illustration of the Residual Swin Transformer.

The schematic illustration of the residual Swin Transformer, composed of four encoding stages, is shown in Figure 4. Given an H×M×3 feature parameter image as input, the patch division operation first splits the input image into $\frac{H}{S}\times \frac{W}{S}$ non-overlapping patches, where S is the patch size. Then, a linear embedding layer projects each patch to a 1×C feature vector. These patch tokens are fed into subsequent stages. The Transformer blocks, together with the patch merging, are referred to as “Stage 1”. The Swin Transformer blocks are applied afterwards for feature transformation. The Swin Transformer blocks are computed as:

(9) $${\hat{z}}^{l}=W\text{-}MSA\left(LN\left(z^{l-1}\right)\right)+z^{l-1},$$

(10) $$z^{l}=MLP\left(LN\left({\hat{z}}^{l}\right)\right)+{\hat{z}}^{l},$$

(11) $${\hat{z}}^{l+1}=SW\text{-}MSA\left(LN\left(z^{l}\right)\right)+z^{l},$$

(12) $$z^{l+1}=MLP\left(LN\left({\hat{z}}^{l+1}\right)\right)+{\hat{z}}^{l+1},$$

where ${\hat{z}}^{l}$ and $z^{l}$ denote the output features of the (S)W-MSA module and the MLP module for block l , respectively; W-MSA and SW-MSA denote window-based multi-head self-attention using regular and shifted window partitioning configurations, respectively. The mechanism of shifted window self-attention reduces computational complexity and allows for efficient long-range interaction among features. The shifting operation ensures overlapping among windows, promoting better integration of local and global context. The patch merging layer performs down-sampling, halving the height and width of the feature map, and doubling the depth. The procedure is repeated twice as “Stage 2” and “Stage 3”, respectively. The Transformer blocks are referred to as “Stage 4”. Thus, the output dimensions of the linear embedding layer and the four stages are: $\frac{H}{S}\times \frac{W}{S}\times C,\;\frac{H}{2S}\times \frac{W}{2S}\times 2C,\;\frac{H}{4S}\times \frac{W}{4S}\times 4C,\;\frac{H}{8S}\times \frac{W}{8S}\times 8C$ , and $\frac{H}{8S}\times \frac{W}{8S}\times 8C$ , respectively. Then, the output of each stage is combined with the feature maps of the previous stages using skip connections. Residual connections facilitate the flow of gradients through the network, enabling deeper networks to learn effectively and retain both low-level and high-level feature information, enhancing semantic richness. A straight line is used here to represent a skip connection. In practice, because the dimensions of different scaled feature maps are different, a certain number of patch merging layers are used in each skip connection for down-sampling (d × 2; here, d is the abbreviation for down-sampling). Finally, the classification results of feature parameter images are outputted through layer norm, global pooling, and fully connected layers. We send m feature parameter images generated by the test video to the Swin Transformer for classification, and judge whether the test video shows fatigue driving behavior according to the proportion of image types.

The details of fatigued driving behavior are reflected in the feature parameter images, as subtle differences in the feature parameters of multiple continuous frames. The residual Swin Transformer module can adaptively adjust the depth and width of the network according to different detail features, providing detailed information from small-scale stages to large-scale ones, which can aggregate feature maps of different sizes and capture fine-grained details to improve recognition accuracy.

### 3.4. Learning Algorithm of FPIRST

The training procedure of the FPIRST model is summarized in Algorithm 1. The key details are illustrated as follows:

(1) In Line 1, the structure of the FPIRST model is constructed. This model consists of SPP-MSFO, MSFLD, the feature parameter matrix and image, and the residual Swin Transformer. The overview of the FPIRST model architecture is shown in Figure 1. The data processing procedure is as follows. Through the getThreeRatioFromvideo (yolo, keyPointModel, videoFile) function, the video is divided into frames, face area detection, and 23 key points on the face location, and the aspect ratios of the left eye, right eye, and mouth are calculated by Formulas (1), (2), and (3), respectively, to form an n×3 feature parameter matrix. The n×3 feature parameter matrix is processed by the Li_n_3toLi224_224 (Li375_3, *n, k, p*) function, and m 224×224 feature parameter matrices are formed. The calculation formula is shown in (6). The feature parameter matrix of 224 × 224 is converted into a feature parameter image ( $X=\left\{X_{1},X_{2},\ldots ,X_{M}\right\}$ ) with a resolution of 224 × 224 by the array2img (dataArray, imgSavePath) function.
(2) In Line 2, the parameters in the FPIRST model are initialized. The parameters include the weight w , bias b , learning rate α , batch size, number of classes, and epochs. These parameters are initialized, as described in Section 4.
(3) In Lines 3–9, the FPIRST model is trained, using forward learning and backward propagation.
(4) In Line 9, model training is completed when the end condition is satisfied. The end conditions include the number of iterations and an early stopping strategy.

**Algorithm 1** Training strategy of FPIRST**Input:** Given 
R 
videos from the HNUFD video dataset, feature parameter image training sample 
$X=\left\{X_{1},X_{2},\ldots ,X_{M}\right\}$
 after data processing and their type labels 
$Y=\left\{Y_{1},Y_{2},\ldots ,Y_{M}\right\}$
.

**Output:** The well-trained model FPIRST.
1: Construct the FPIRST method shown in Figure 1;
2: Initialize the parameters;
3: **Repeat**
4:   Randomly select a batch of instances 
$X_{b}$
 from 
X
;
5:   Forward learn training samples through the FPIST model;
6:   Compute the error 
$L\left(\theta \right)$
 by 
$L\left(\theta \right)=\|y_{t}-{\hat{y}}_{t}{\|}_{2}^{2};$

7:   Propagate the error back through FPIST and update the parameters;

8:   Find 
θ
 by minimizing 
$L\left(\theta \right)$
 with 
$X_{b}$
;
9: **Until** the end condition is satisfied.


## 4. Experiments

In this section, we evaluated the performance of the proposed fatigue driving recognition method from three aspects: (1) ablation study of the FPIRST method and the residual Swin Transformer model, (2) comparison of the accuracy of the proposed method under different numbers of sliding frames, and (3) comparison with other methods.

### 4.1. Setting

#### 4.1.1. Experimental Conditions

The experiments were conducted on a 64-bit Ubuntu 20.04 platform with an Intel x299 Core i9-10900X CPU @3.7 GH (Santa Clara, CA, USA), NVIDIA GeForce RTX 3090 (Santa Clara, CA, USA), and 48 GB memory. Python 3.8 language and PyTorch 1.12.1 framework were used. Parameter initialization in the forward pass and backward fine-tuning is important for model training. In this study, the weights between layers were initialized randomly and obeyed a uniform distribution. All biases were initialized as zero. Model optimization used stochastic gradient descent with a momentum of 0.9, learning rate of 0.0001, batch size of 8, epochs of 100, and 2 classes. Each baseline network architecture was trained with an identical optimization scheme. The experimental conditions and parameter settings are shown in Table 1.

#### 4.1.2. Evaluation Metrics

Accuracy is an important index for measuring fatigue driving recognition performance. Its definition is shown in Equation (13):

(13) $$\mathrm{Accuracy}=\frac{\mathrm{TP}+\mathrm{TN}}{\mathrm{TP}+\mathrm{TN}+\mathrm{FP}+\mathrm{FN}}$$

where TP is the number of true positives, TN is the number of true negatives, FN is the number of false negatives, and FP is the number of false positives.

### 4.2. Datasets

The Hunan University Fatigue Driving dataset is referred to as the “HNUFD” dataset. The HNUFD video dataset contains 26 male drivers and 15 female drivers, including glasses, no glasses, different hairstyles, different costumes, and drivers of different ages. In the video dataset, most participants shot five types of driving videos, namely dozing, yawning, normal, closed-mouth, and talking. The video dataset contained 341 videos, each 15 s long, including 202 videos in the training set and 139 videos in the test set. These videos were collected using infrared cameras, with a resolution of 1920 × 1080 pixels, 24-bit depth, and 25 frames per second. These videos were recorded under different conditions, such as sunny, cloudy, rainy days, and evenings to reflect different lighting conditions. To reflect real driving environments, we recorded driving videos.

### 4.3. Ablation Studies

We performed ablation experiments on the FPIRST method and the residual Swin Transformer model to demonstrate the effectiveness of the proposed method.

#### 4.3.1. Architecture of the FPIRST Method

We conducted experiments using a combination of different configurations, such as framed image modules (FIs), face region image modules (FRIs), feature parameter image modules (FPIs), Swin Transformer modules (STs), and residual Swin Transformer modules (RSTs), which proved the effectiveness of our proposed FPIRST method. In this ablation study, we used the HNUFD video dataset and the base architecture of Swin-B.

We compared six combined variants: FI + ST, FI + FRI + ST, FI + FRI + FPI + ST, FI + RST, FI + FRI + RST, and FI + FRI + FPI + RST. The results are shown in Table 2. Accuracy increases with the use of FI, FRI, and FPI, and the accuracy of RST is higher than that of ST. When FI + FRI + FPI + RST is combined, the performance of the model is the best, and the accuracy rate is 96.4029%. The framed image includes information about the driver and the environment inside the car in a single-frame image. The face region image contains the face area information in the single-frame image. The feature parameter image not only reflects eyes and mouth information in each frame image but also contains time information from multiple continuous frames. Fatigue driving behaviors mainly involve the movements of the eyes and mouth and are persistent, which is why the feature parameter image is used as the input image for the RST module, thus increasing accuracy.

#### 4.3.2. Residual Swin Transformer Model

We used feature parameter images generated by 202 training videos when the sliding frame number was 75 as the training image dataset of the model. We used the RST, full-scale residual Swin Transformer module (FSRST) [25], and ST to obtain three training models. Then, we tested the accuracy of each model using 139 test videos, as shown in Table 3.

Table 2 shows that the RST model has higher accuracy than the FSRST and ST models because it combines the output from each stage with the feature diagram of the previous stage by using skip connections, thus allowing the network to adaptively adjust its depth and width according to features with different details. Fine-grained details are captured, and recognition accuracy is improved by aggregating feature maps of different sizes.

### 4.4. Comparison of the Accuracy of the Proposed Method under Different Sliding Frame Numbers

We first built a training dataset for the residual Swin Transformer, and then trained the residual Swin Transformer model. The accuracy of the proposed method was compared under different numbers of sliding frames.

#### 4.4.1. Building A Training Image Dataset for the Residual Swin Transformer Model 

**Figure 5:**
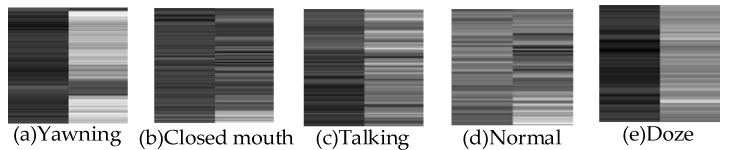
Feature parameter images in the created Residual Swin Transformer image dataset.

First, each video in the HNUFD video dataset was divided into n images, and the face region of each image was obtained by the SPP-MSFO detection module. The MSFLD model was used to locate the coordinates of 23 key points in the face region of each image; the aspect ratio of the left eye, right eye, and mouth was calculated according to the key points, and an n×3 feature parameter matrix was formed for each video. Then, we repeated the aspect ratios of the left eye, right eye, and mouth in the n×3 feature parameter matrix 56, 56, and 112 times, respectively, turning the matrix into an n×224 feature parameter matrix. By sliding k frames each time ( k was 25, 50, 75, 100, and 125, respectively,) the n×224 matrix was turned into m 224×224 feature parameter matrices. Finally, we converted each 224 × 224 matrix into a feature parameter image. After processing, each video became m feature parameter images. The 202 training videos in the HNUFD video dataset created m×202 feature parameter images. We labeled the images produced by videos of dozing and yawning as “fatigue” feature parameter images, and those produced by videos of normal, closed-mouth, and talking as “normal” feature parameter images. In this way, we built the training image dataset of the Swin Transformer. Feature parameter images in the created residual Swin Transformer image dataset are shown in Figure 5.

#### 4.4.2. Training the Residual Swin Transformer Model 

Based on the constructed training image dataset of the residual Swin Transformer, we trained the residual Swin Transformer model with epochs of 100, a batch size of 8, a learning rate of 0.0001, and a classification number of 2. The number of sliding frames was different, which is why the content and number of feature parameter images in the created training data set were also different. Therefore, when different sliding frames were selected, the trained residual Swin Transformer model was different. In our experiments, the number of sliding frames was set to 25 (1 s), 50 (2 s), 75 (3 s), 100 (4 s), and 125 (5 s), corresponding to five trained residual Swin Transformer models. The time to train a model was 1300 s, the time for forward propagation was 0.0042 s, and the parameters of the model were 2,770,306.

#### 4.4.3. Comparing the Accuracy of the Proposed Method under Different Numbers of Sliding Frames

On the basis of the 139 test videos in the HNUFD video dataset, five trained residual Swin Transformer models were used to test the accuracy of the proposed method with sliding frames of 25 (1 s), 50 (2), 75 (3 s), 100 (4 s), and 125 (5 s), respectively. According to the data in Table 4, when the number of sliding frames is 75, the accuracy rate of the proposed method is 96.512%, which is the highest.

### 4.5. Comparison with Other Methods

The proposed method was compared with the threshold method [6], SVM [8], LSTM [9], and Bi-LSTM [10]. The detailed description of each baseline is as follows:

Threshold method [6]: This fatigue driving behavior recognition method combines adaptive threshold and statistical threshold, in which the aspect ratio threshold of eyes is taken from the maximum value of the adaptation threshold and the statistical threshold, and the aspect ratio threshold of the mouth is taken from the minimum value of the adaptation threshold and the statistical threshold. The adaptive threshold is dynamic and obtained by calculating the eye and mouth aspect ratios in the first 30 frames of each test video, solving the problem of differences in the aspect ratios of the eyes and mouths of different drivers. The statistical threshold is fixed and obtained by calculating the average of the aspect ratio of eyes and mouth for different driving behavior types and drivers, to solve the problem of the adaptive threshold possibly having a low aspect ratio of eyes and a high aspect ratio of mouth when testing the videos of fatigue driving behavior.

SVM [8]: SVM is a general linear classifier that is a binary classification of data based on supervised learning methods. The main idea of SVM is to find an ultra-flat plane that divides samples into two categories and classifies the largest interval. SVM can be used for fatigue classification. First, two sets of fatigue and non-fatigue vectors are marked. Then, an optimal division of ultra-flat planes is obtained to divide these two sets of vectors on both sides so that the distance between the support vector is as far as possible. Finally, the classification results are obtained. The advantage of SVM is that it can deal with high-dimensional data problems and is still valid even if the data dimension is larger than the number of samples. SVM does not need to rely on the whole dataset and has strong geralization ability. The disadvantage of SVM is that it is more sensitive to missing data, and sometimes, finding a suitable nuclear function to perform data dimensions is difficult.

LSTM [9]: LSTM is a type of gate control circulating neural network. The LSTM model has the ability to forget and remember certain information. These capabilities are achieved through gate control units. The LSTM unit includes an input gate, forget gate, output gate, and a state unit. The input gate controls which input information should be stored in the state unit. The forget gate controls which information should be forgotten or remembered. The output gate controls which information in the state unit should be output to the next time step. These gates control the inflow and outflow of information by learning weights and long-term dependencies in the input sequence, and remembering important information over longer periods. Fatigue driving is continuous over time, and the fatigue feature vector of continuous frames is stitched into a time feature sequence and entered into the LSTM network to determine whether fatigue driving is occurring.

Bi-LSTM [10]: The Bi-LSTM network is a model that combines the forward LSTM and the backward LSTM. LSTM can capture dependencies in the input sequence but it cannot encode information from back to front. Bi-LSTM can use not only historical sequence information but also future information. Bi-LSTM is compared to LSTM for better extraction of feature information and sequence information. The use of Bi-LSTM in fatigue driving detection can better capture front and back dependencies in the input sequence, realizing the integration of feature information and time sequence information, and improve the accuracy of fatigue driving recognition.

This experiment is conducted on the HNUFD test video dataset to compare the accuracy of the proposed method with the above-mentioned baselines. Table 5 shows the comparison results on the HNUFD test video dataset for fatigue driving behavior recognition. The accuracy of our proposed method is 96.4029%, which is higher than that of the other five methods. These results indicate that the proposed FPIRST is more effective than the state-of-the-art method in real scenes for fatigue driving behavior recognition.

**Figure 6:**
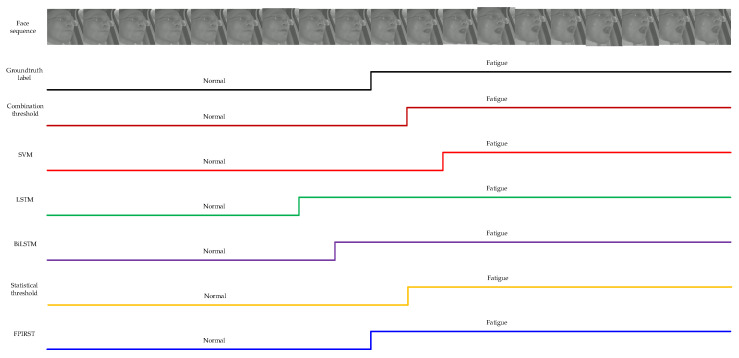
The illustration of identification results of various fatigue driving behavior methods.

**Figure 7:**
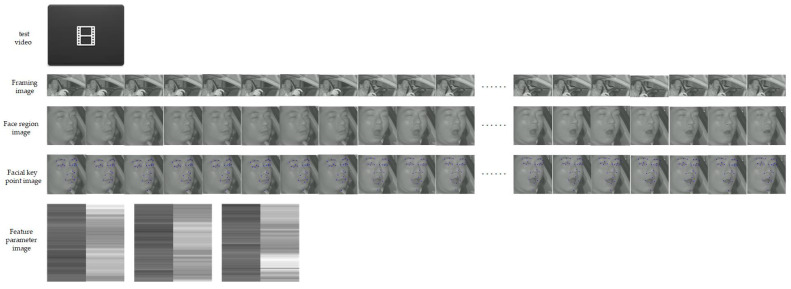
Visual result diagrams of each module of the FPIRST method.

We extracted 19 continuous facial region images from a test video, and then identified them using the above different fatigue driving behavior methods. The results are shown in Figure 6. We conducted visual experiments on each module of the proposed FPIRST method, and the results are shown in Figure 7.

## 5. Conclusions

In this study, we presented a fatigue driving recognition method based on feature parameter images and a residual Swin Transformer. The proposed method is composed of face region detection, facial key point location, fatigue parameter feature matrix construction, fatigue parameter feature images, and a residual Swin Transformer network. Fatigue is a continuous behavior. If results depend only on the eyes and mouth of a single-frame image, an error judgment can be made easily. Therefore, we built the fatigue parameter feature matrix using multiple continuous frames and converted the matrix into a fatigue parameter feature image. We sent this image to the residual Swin Transformer network to determine fatigue driving. The accuracy of the proposed method is 96.4029% on the HNUFD test video dataset. However, the effect of the proposed method in fatigue driving recognition in complex scenes, such as when drivers wear glasses, is not ideal.

In the future, we will conduct research on the following aspects: (1) we will consider data, such as head posture and facial expressions, to expand the fatigue parameter features and enhance the robustness of the system; (2) during fatigue driving behavior detection under complex driving environments, such as when the car enters a tunnel, drivers with glasses, or driving in high-altitude environments, the physiological and behavioral performance of the driver may be different. We will dynamically adjust the standards of fatigue detection according to the differences in the driving environment to improve the accuracy of fatigue driving detection; (3) we will study a lightweight fatigue driving behavior detection algorithm to meet commercial real-time requirements.

## Figures and Tables

**Table 1:** Experimental conditions and parameter settings.

	Device or Parameter	Details
Experimental conditions	CPU (Santa Clara, CA, USA)	Intel x299 Core i9-10900X @3.7 GH
	Memory	32 G
	Motherboard	Z690 DDR4
	GPU (Santa Clara, CA, USA)	NVIDIA RTX 3090 48 G
	Platform	64-bit Ubuntu
parameter settings	Language	Python
	Framework	PyTorch
	Learning rate	0.0001
	Batch size	8
	Epoch	100

**Table 2:** Effect of using different configurations on fatigue driving recognition using the HNUFD test video dataset.

Formulation	Accuracy (%)
FI + ST	82.7338
FI + FRI + ST	82.7338
FI + FRI + FPI + ST	84.8921
FI + RST	82.7338
FI + FRI + RST	87.0504
FI + FRI + FPI + RST (Proposed)	96.4029

**Table 3:** Effect of using different architecture variants of the Swin Transformer network on fatigue driving recognition using the HNUFD test video dataset.

Model	Accuracy (%)
Swin Transformer [20]	84.8921
Full-scale Residual Swin Transformer [21]	84.8921
Residual Swin Transformer	96.4029

**Table 4:** Accuracy of the proposed method under different sliding frame numbers.

Frame Numbers	Accuracy (%)
25 (1 s)	84.8921
50 (2 s)	84.1727
75 (3 s)	96.4029
100 (4 s)	87.7698
125 (5 s)	84.1727

**Table 5:** Comparison with existing methods on the HNUFD test video dataset for fatigue driving behavior recognition.

Methodology	Accuracy (%)	Model Size (MiB)	Test Time (s)
Combination threshold [6]	86.3309	-	25.6132
SVM [8]	74.1007	0.211	25.9192
LSTM [9]	82.7338	0.292	29.9493
BiLSTM [10]	85.6115	3.2	33.9743
Statistical threshold [12]	90.6475	-	25.6205
FPIRST	96.4029	343.1	26.7026

## Data Availability

The relevant data of this paper can be accessed by contacting Weichuxiao@hnu.edu.cn.
